# On species delimitation: Yet another lemur species or just genetic variation?

**DOI:** 10.1186/1471-2148-11-216

**Published:** 2011-07-21

**Authors:** Matthias Markolf, Markus Brameier, Peter M Kappeler

**Affiliations:** 1 Behavioral Ecology and Sociobiology Unit, German Primate Center, Kellnerweg 4, 37077 Göttingen, Germany; 2 Department of Primate Genetics, German Primate Center, Kellnerweg 4, 37077 Göttingen, Germany; 3 Department of Sociobiology/Anthropology, University of Göttingen, Kellnerweg 6, 37077 Göttingen, Germany

## Abstract

**Background:**

Although most taxonomists agree that species are independently evolving metapopulation lineages that should be delimited with several kinds of data, the taxonomic practice in Malagasy primates (Lemuriformes) looks quite different. Several recently described lemur species are based solely on evidence of genetic distance and diagnostic characters of mitochondrial DNA sequences sampled from a few individuals per location. Here we explore the validity of this procedure for species delimitation in lemurs using published sequence data.

**Results:**

We show that genetic distance estimates and *Population Aggregation Analysis *(PAA) are inappropriate for species delimitation in this group of primates. Intra- and interspecific genetic distances overlapped in 14 of 17 cases independent of the genetic marker used. A simulation of a fictive taxonomic study indicated that for the mitochondrial D-loop the minimum required number of individuals sampled per location is 10 in order to avoid false positives via PAA.

**Conclusions:**

Genetic distances estimates and PAA alone should not be used for species delimitation in lemurs. Instead, several nuclear and sex-specific loci should be considered and combined with other data sets from morphology, ecology or behavior. Independent of the data source, sampling should be done in a way to ensure a quantitative comparison of intra- and interspecific variation of the taxa in question. The results of our study also indicate that several of the recently described lemur species should be reevaluated with additional data and that the number of good species among the currently known taxa is probably lower than currently assumed.

## Background

Species are the fundamental units of evolutionary biology as they define the entities that are studied and compared in every field of biology [1]. Moreover, they are the currency for biodiversity classification of geographic regions, and are therefore used to define regions of conservation priority, so-called biological hotspots [2,3]. Despite the central importance of species, there is no general agreement about what a species is, and the 'species problem' is one of the most discussed topics in evolutionary biology [4-6].

An overview of species concepts is beyond the scope of this article, but it should be emphasized that the discussion has shifted away from the philosophical and conceptual questions towards a more pragmatic approach in recent years [7,8]. De Quieroz [1] argued that all modern species definitions are variations on the same general linage concept of species, because these definitions equate species either explicitly or implicitly with segments of population level evolutionary lineages [1,9-11].

Adopting a concept of species as population level lineages will not solve the problems related to species delimitation in practice, but there would no longer be a discussion of the species concept [1]. In doing so, the concept of species and the question how we recognize a species in practice are encapsulated [12], which means that no single property is necessary to be considered crucial, as is reproductive isolation for the Biological Species Concept (BSC) or a phylogenetically distinct cluster for the Phylogenetic Species Concept (PSC), because every single criterion is likely to fail or to yield ambiguous results [6,11,13]. As empahasized by Ernst Mayr [14], species should therefore be delimited with different datasets (criteria) [3,10,15-19]. In practice, morphological and molecular approaches are mutually informative [20] and often feasible.

The recent taxonomic practice in the primates of Madagascar (Lemuriformes) looks quite different for the most part. Tattersall [21] recently questioned whether the dramatic increase of recognized lemur species in recent years is due to previously unnoticed cryptic diversity or to taxonomic inflation. In 1982, he counted 36 lemur species, whereas in 2007 already 83 species were recognized. This is an increase of 1.88 lemur species per year over 25 years, which is partly due to the fact that small, nocturnal animals were actually being captured for the first time, that research effort has increased, that remote forests have been visited and that new molecular techniques have become available. In 2011, the count is currently at 101 species [22], which means that the rate of new species descriptions more than doubled (to 4.5 species per year) in the last 5 years alone. Are we still unraveling cryptic taxonomic diversity or has the use of particular methods or criteria kindled taxonomic inflation? Because Tattersall's question seemed to have been largely ignored, we re-visit this problem, using quantitative genetic methods to scrutinize methods and concepts used to describe new lemur taxa.

It is particularly striking that several recent taxonomic studies of lemurs are based almost exclusively on evidence from mitochondrial DNA (but see [23-28]. Even where morphometric data were available, they were not analyzed statistically [29-31]. Specifically, a relatively small number of individuals per location were typically sampled in formerly uninvestigated areas. Mitochondrial DNA was then sequenced and compared with previously published data. If the sampled individuals clustered together in a phylogenetic tree and interspecific genetic distances between the new and other taxa were in the range of previous published interspecific distances within the genus under study, and if additional diagnostic sites could be determined via Population Aggregation Analysis (PAA) [32], a new species was proposed and eventually described.

Genetic distances are valid tools for taxonomy because sequences of different organizational levels (e.g. within species, within genera, within families) exhibit different amounts of divergence, which do not overlap and create a gap [33]. This gap can be used as an objective threshold for a species boundary. One indispensable prerequisite for this procedure is to calculate genetic distances at both levels of organization (within and between species) in order to identify the gap. This was often not the case in lemurs (e.g. see [29-31]). For example, comparisons of intraspecific levels of divergence for populations of *Microcebus *[34] and *Lepilemur *[35] were based on as few as 3 individuals (*M. bongolavenesis)*, but it is not known whether this is sufficient for a representative characterization of the existing intraspecific variation. Similarly, [36] divergence estimates of the D-loop of 3.7% between *M. margotmarshae *and *M. mamiratra *were used in identifying the former as a new species. This approach needs to be reconciled with the observation of Fredsted et al. [37], who found genetic divergences of up to 8.2% among potentially interbreeding individuals of *Microcebus murinus *within an area of 3 km^2 ^of continuous forest. In light of these overlapping levels of genetic variance within and between taxa, the question arises on which criteria species delimitations should be based and which sample sizes are likely to be sufficient to identify true differences.

The problem of an appropriate sample size is also relevant for PAA, a method frequently used to support inferences about the existence of new taxa in combination with the PSC (e.g. [30,31,34-36]). PAA compares homologous sequences drawn from different populations. A position (base in DNA sequence) that is fixed (i.e. fully conserved) in one population, but has a different state (base) compared to another population is treated as diagnostic site or character. Although it is known that PAA is prone to small sample sizes [38,39], we also asked how PAA would be influenced by sample size, using a simulation with data from a real population of *Microcebus*, a genus with particularly many recently described new species.

The aims of this study were, therefore, to use the publicly available information about genetic variation from different lemur taxa to identify typical levels of intra- and interspecific genetic variation at loci commonly used in species delimitation and to determine minimal reliable sample sizes for these types of analyses. It is explicitly not our intention to single out particular studies for criticism. We know from personal experience that field work in Madagascar can be extremely difficult, that some species live at low densities and or high up in the canopy, making access to a desirable number of samples very difficult. We also realize (but do personally not endorse) the view that sacrificing potentially rare animals for proper description and deposition in an accessible museum is ethically challenging for some; a fact that may also contribute to false positives and an inflation of species numbers. Finally, it can also be argued that assigning species status to a potentially endangered taxon is a politically justified strategy in order to achieve maximal preemptive conservation effects because extinction cannot be reversed. This approach will also favor splitting over lumping and contribute to an increase in species numbers. All these aspects and problems at the interface of sound scientific procedures, practical difficulties of fieldwork and conservation politics can benefit from sound empirical criteria, which we hope to contribute with these analyses.

## Results

### Genetic distances

**Figure 1:**
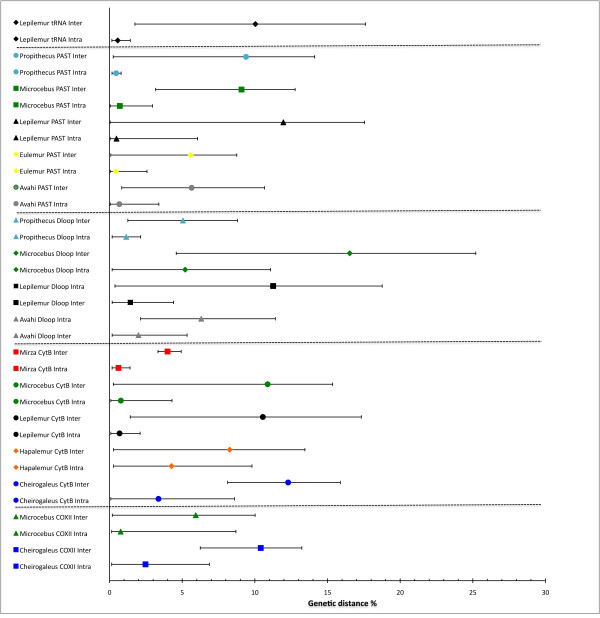
**Genetic distance plot**. x- axis = Genetic distance in %; y- axis = lemur genera and analyzed marker with unique identifier. Interspecific distances per taxa are plotted above intraspecific. Plots are grouped by marker.

Intra- and interspecific genetic distances are plotted pair-wise for each taxon and marker in Figure 1. Only the genetic distances of *Lepilemur *for the tRNA marker, the *Microcebus *distances for the PAST fragment [40] and the cytochrome B distances for *Mirza *show no overlap. All other pair-wise plots show more or less overlap of intra- and interspecific genetic distances. In several cases the smallest interspecific value even exceeds the lower level of intraspecific variation. None of the different markers show a superior performance over different genera. *Lepilemur *and *Microcebus *exhibit the highest intra- and interspecific variation for all markers.

### PAA Simulation

**Figure 2:**
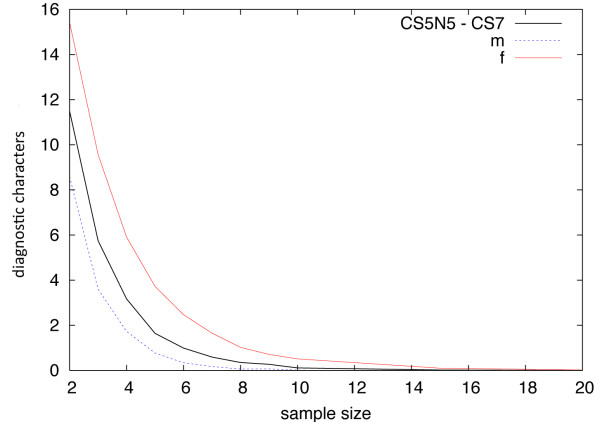
**Plot of mean diagnostic characters per sample size**. X-axis = samples drawn from each population, Y-axis = diagnostic characters (a site in a DNA sequence of a population that has a fixed but different state as in another population), CS7 = Population 1, CS5N5 = Population 2, males = blue, females = red; 2, 4, 6, ..., 20 Individuals were randomly drawn 10,000 times.

The simulation of diagnostic characters (Figure 2) revealed that two individuals drawn from a population lead to 11-12 diagnostic sites that would argue for a separation into two species. The curve describing the relationship between sample size and the number of diagnostic sites drops relatively fast. However, 10 individuals randomly drawn from each population can still occasionally lead to the identification of a diagnostic character as the curve has not reached 0 yet. What is also evident is that sampling only females is much more likely to produce diagnostic sites than sampling only males. Random sampling of 8 females per population still results in one diagnostic character, on average, arguing for separation into two species according to the PSC.

## Discussion

### Genetic distance

The comparison of intra- and interspecific distances across several lemur genera and markers revealed that none of the commonly used markers are generally suitable for distance-based species delimitation in this taxon. One possible error in our estimation could be the wrong assignment of an individual to a certain species, because of changing taxonomy. However, we checked affiliation several times in all cases and used the most recent publication referring to the sequence in question.

The overlap of intra- and interspecific distances in most cases is best explained by paraphyly and polyphyly of the mitochondrial DNA [41] of the relevant taxa. For example, the overlap of *Avahi *distance estimates for the D-loop and PAST fragment is due to paraphyly of *Avahi peyriasi *[29,42]. Three types of *Avahi peyriasi *are distinguished. The fact that all of them actually occur at one site (Ranomafana) indicates that the taxonomy of the south-eastern *Avahi *taxa (*A. peyrierasi, A. betsileo, A. ramanantsoavanai, A, meridionalis*) is highly questionable and should be revised.

The same problem applies to *Eulemur fulvus*, which was also paraphyletic for the PAST fragment [40]. *Hapalemur aloatrensis *is not distinguishable from *Hapalemur griseus *on a molecular basis. This, and the paraphyly of *Hapalemur griseus *subspecies, leads to the observed overlap in cytochrome B [43,44]. Interspecific distances of *Lepilemur *(D-loop; PAST) are as small as the lower limit of intraspecific distances. Zinner et al. [45] already questioned the existence of *L. mittermeieri *and *L. tymerlachsonorum*. Where intraspecific divergence reaches high levels, e.g. 8% in *Microcebus *for COX II, we can expect that more species are going to be described if this locus is being used. Indeed, these 8% are caused by individuals from Bemanasy, which seem to form an independently evolving linage [25].

Another factor influencing the overlap of intra- and interspecific distances might be the geographical distribution of different taxa. Whereas some taxa like *M. murinus *are widespread (but see Weisrock et al. 2010), others, such as *M. tavaratra *occur only in very restricted areas [22].

Whatever the explanation for the overlap of intra and interspecific distances in different taxa, the present analysis indicates that a constant "threshold species delimitation", as it is used in barcoding approaches, cannot be recommended [46].

### PAA simulation

For the present simulation, we used as diagnostic characters only those sites that are fixed and different between populations. Sites that are variable within populations, but different between populations are sometimes also referred to as being diagnostic attributes [47,48], and would lead to an even higher number of diagnostic characters.

Our simulation showed that sampling fewer than 10 individuals can falsely lead to diagnostic characters and to an argument for identifying a new species under the PSC. The number of published diagnostics characters for several recently newly described lemur species for the mitochondrial D-loop are far below 10 (e.g. [29]). Because this analysis was focused on the highly variable mitochondrial D-loop, this value should not be used as a general guideline for taxonomic sampling. For less polymorphic markers, such as cytochrome b for example, the curve would probably need fewer individuals to reach zero. However, to establish a general sampling threshold the same analysis ought to be repeated for several different markers and populations. Walsh [38] estimated necessary sampling values of > 50 individuals in order to perform well with PAA. Wiens and Servidio [39] even argued that hundreds and thousands of individuals would be necessary to identify diagnostic characters that are valid for the species boundary. This is unpractical and impossible for most taxonomic studies, however. Hence, other species delimitation methods should be favored and are discussed below.

Finally, the simulation revealed a clear difference between males and females. Because of its uniparental inheritance and male-biased dispersal in *Microcebus*, mitochondrial DNA exhibits necessarily higher divergence between populations [49]. That does not mean that there is no genetic exchange via males, however. Gene flow is an important feature of species, especially in introgressed species. Therefore, genetic markers with high levels of gene flow in the dispersing sex should be more effective for species delimitation [50].

### How to delimit species?

We have argued that sole analysis of uniparentally inherited genomes, like mtDNA, is not sufficient to delimit species, as it does not realistically reflect the population history [41]. On the other hand, sequencing other parts of the genome revealed that gene trees can differ substantially between different loci [51-54] because each locus has its own evolutionary history [55]. These differences between loci can challenge the delimitation of species via nuclear DNA, but can also be used to draw inferences about population size and subdivision, gene flow and hybridization [53], all of which play a role in generating new taxa and biodiversity. The use of multiple loci including nuclear and sex-specific markers in studying the evolutionary history of populations has already been applied in several other organsims [51,55-58] apart from lemurs (for exceptions see [23-25]), and is highly recommended to obtain a realistic picture of the population history [59] and to adequately describe phylogenies at and below the species level [60]. Recent advances in sequencing technology provide the possibility for multilocus analyses, even of non-model species (for lemurs see [61]). The use of multilocus sequence data requires different statistical procedures, which become more and more sophisticated. Likelihood and Bayesian summary statistics are now commonly used in phylogeographic and phylogenetic inference and replace older methods that rely on single gene trees [62,63].

Using Bayesian structure analysis [64] and the Genealogical Sorting Index (GSI) [65] Weisrock et al. [25] confirmed the high number of *Microcebus *species using several nuclear markers, although species were not reciprocally monophyletic. In contrast, using also several nuclear markers in combination with morphological data, Groeneveld et al. [23,24] reduced the number of *Cheirogaleus *species from 7 to 4, indicating the suitability to delimit species with several types of information [3,10,15-18,66-68]. For example, morphologically distinct mouse lemurs [26] could be comfirmed as separate species with genetic data [27]. Similarly, Zimmermann [69] and Nietsch [70] have emphasized the suitability of vocalizations for species delimitation in non-human primates, and this type of data has been used to clarify the taxonomy of tarsiers, for example [71]. Whatever these data might be, genetic samples, morphological measurements or other types of data should be sampled in a way that intraspecific variation can be assessed and compared to interspecific variation before new species are described.

Why lemur taxonomists have not used the above-mentioned criteria to delimit species is only speculative, but one reason might have been that collecting high quality samples for DNA analyses from many individuals is anything but easy. Furthermore, the methods to extract nuclear DNA from low quality samples such as fecal or museum samples and sequencing those at low costs as well as nuclear primers were only recently developed [61]. Finally, from a conservation perspective, the urgent need to protect several highly threatened areas in Madagascar may have favored splitting species over lumping as well.

## Conclusions

We conclude that PAA and genetic distances are inappropriate singular methods to delimit lemur species. Furthermore, we encourage the use of several nuclear and sex- specific genetic loci as well as the combination of different datasets for species delimitation. Populations that are considered to be different species should be sampled in a way that intraspecific variation can be compared with interspecific variation. Recently described lemur species should be critically re-evaluated, and we predict a taxonomic deflation for several genera.

## Methods

### Genetic distances

We searched the NCBI database for published lemur sequences and downloaded those in the application Geneious Pro (version 4.8.5). Sequences were grouped by genus and sub-grouped by sequenced loci. Taxonomic identity of each sequence was either based on the publication or on locality, if taxonomy was likely to have changed over years. Sequences were aligned using the ClustalW plugin in Geneious and afterwards checked by eye. Distances were estimated using the software MEGA [72]. We calculated p-distances, as it is the mostly used method in previous lemur publications and report distances as percentage genetic distances. Gaps or different length of sequences were not used for calculations as we chose the pair-wise deletion option in MEGA.

We calculated genetic distances within species (intraspecific) and between species (interspecific). Values were exported to Excel to process and to visualize distances. Afterwards we plotted the mean and the range to the lowest and highest value of intra- and interspecific distances per marker and taxon.

### Simulation

To simulate the impact of sample size to the results of PAA on the number of species, we used one of the best-studied mouse lemur population at Kirindy Forest. The published dataset consists of 202 different gray mouse lemur individuals (*Microcebus murinus*), which showed 22 haplotypes for the mitochondrial D-loop [37]. All sequences were aligned and cut to equal length (529bp) The gray mouse lemur population at Kirindy showed significant genetic structure between 3 local study sites (CS5, CS7 and N5), which are 2-3 km apart (see Fredsted et al. [37] for details of the study area). This substructure was used for the simulation as different sampling areas for a fictive taxonomic study. We divided the population into two sampling areas (CS5 and N5 vs CS7), including approximately the same number of individuals in each population.

Afterwards 2, 4, 6, ...20 sequences were drawn randomly from each population 10,000 times for the entire dataset and for males and females separately. After each step the number of diagnostic characters were determined and the mean was plotted against the number of sequences drawn from each population. Simulations were done using PERL (PERL script can be received by request from the authors).

## Competing interests

The authors declare that they have no competing interests.

## Authors' contributions

MM and PMK conceived the study and wrote the paper. MM and MB analyzed data and conducted the simulation. All authors have read and approved the final manuscript.
